# Bigger isn't always better: Challenging assumptions about the associations between diapause, body weight, and overwintering survival

**DOI:** 10.1002/ece3.11511

**Published:** 2024-06-03

**Authors:** Clancy A. Short, Jared L. Walters, Daniel A. Hahn

**Affiliations:** ^1^ Department of Entomology and Nematology The University of Florida Gainesville Florida USA

**Keywords:** body weight, diapause, dormancy, feeding, life history, nutrition, overwintering, starvation

## Abstract

During the winter, animals face limited food availability. Many animals enter dormancy to reduce their winter energy expenditure. Most insects spend the winter in diapause, a state of programmed dormancy. It is often assumed that diapausing insects need nutrient stores to fuel their many months of basal metabolism and must grow heavier than their non‐diapause‐programmed counterparts. However, the extent to which food limitation affects body weight during overwintering preparation as well as the likelihood and duration of diapause remains unclear. We limited the duration of the feeding period and thus the total quantity of food available to diapause‐destined larvae of the pupal‐diapausing flesh fly, *Sarcophaga crassipalpis*, to test how food limitation affects body weight in the context of diapause programming. We also tested the extent to which food deprivation and body weight affect the likelihood and duration of diapause. We hypothesized that diapause‐destined larvae grow more quickly and pupariate at a heavier body weight than non‐diapause larvae. We also hypothesized that body weight is more dramatically reduced by food limitations when a larva is programmed for diapause. Finally, we hypothesized that larvae with lighter body weight (i.e., food limited) are less likely to enter pupal diapause and also stay in diapause for a shorter duration than heavier, well‐fed, individuals. Contrary to our hypotheses that diapausing insects are heavier than their non‐diapausing counterparts, we found diapausing pupae weighed less than non‐diapausing pupae, especially when larvae received limited food. We found light pupae did not abort their diapause program. In both diapausing and non‐diapausing pupae, body weight was positively correlated with simulated winter survival. However, above a weight threshold, body weight no longer affected simulated winter survival in diapausing pupae. Contrary to our predictions and the general consensus in much of the diapause literature, we also found that lighter pupae stayed in diapause longer than heavier pupae. Overall, our results challenge the precept that body weight and diapause are positively associated. The relationship between body weight and diapause is complex and may be affected by the availability of food before and after winter, the availability of high‐quality overwintering sites, and the life history of a particular insect.

## Graphical abstract

Our findings challenge the positive association between body weight and diapause (dormancy) phenotypes in the model flesh fly, *Sarcophaga crassipalpis*. Nutrient limitation alters the relationship between body weight and diapause phenotypes, suggesting that the relationships between body‐weight and phenotypes need to be considered within ecological contexts.

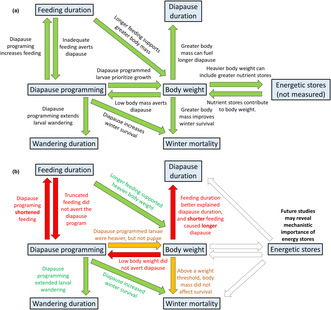

## 1 INTRODUCTION

Harsh environmental conditions encountered during the winter pose a major threat to many temperate zone insects. Andrewartha and Birch (1986) propose that thermal limits experienced during winter set the range limits for many animals, including insects, and this concept has been a pervasive theme of the literature on range limits ever since (Phillips et al., 2010; Williams et al., 2015). Not only do cold winter temperatures inflict abiotic stress upon an animal, but food sources are often scarce or absent during the winter. In preparation for winter, many mammals enter hibernation, a state of reduction of locomotion, body temperature, and metabolic rate (Lyman & Chatfield, 1955). Similarly, many insect species enter a programmed state of dormancy called diapause to avoid the stressful conditions of winter and synchronize their lifecycle with favorable environmental conditions and food availability (Denlinger, 2022). Like a hibernating mammal, insects often drastically reduce their metabolic rate during diapause, but the long duration of diapause, usually in the absence of feeding, often leads to substantial depletion of nutrient reserves (Hahn & Denlinger, 2007, 2011; Lehmann et al., 2016; Roberts & Williams, 2022; Sadakiyo & Ishihara, 2012; Sinclair, 2015). Enhanced stress hardiness is often also part of the diapause developmental program, and insects can accumulate cryoprotectants like glycerol, sorbitol, and heat shock proteins, among others, prior to winter, creating another nutrient cost associated with diapause (Danks, 2006; Hodkova & Hodek, 2004; Rinehart et al., 2000). Additionally, insects may require nutrients to repair tissue damage caused by adverse temperatures during winter (Colinet et al., 2007; Koštál & Tollarova‐Borovanska, 2009; MacMillan et al., 2015; Štětina et al., 2018) and to support post‐winter activities like metamorphosis or dispersal (Chaplin & Wells, 1982; Dhillon & Hasan, 2018). Given the nutrient costs associated with diapause, one might predict that insects should increase their feeding to accumulate nutrient reserves prior to diapause, and thus become heavier than their non‐diapausing counterparts as part of the diapause preparatory program (Danks, 1987; Denlinger, 2022; Hahn & Denlinger, 2011; Koštál, 2006; Short & Hahn, 2023; Sinclair, 2015; Tauber et al., 1986). In mammals, fattening to prepare for hibernation is also considered a general rule, best exemplified by grizzly bears, *Ursus arctos*, accumulating 90 kg of fat to prepare for their hibernation (Hilderbrand et al., 2000). Growing heavier and fattening can also allow an animal to use its stores relatively more efficiently because the relative resting metabolic rate decreases as body weight increases (Niven & Scharlemann, 2005). In other words, 1 kg of fat can fuel the resting metabolism of a 100 kg animal longer than 1 g of fat can fuel the resting metabolism of a 100 g animal. However, insects and other animals often prepare for diapause or hibernation as the growing season comes to an end and food sources become scarce, so additional feeding may require dangerous foraging or increase the risk that an insect succumbs to the first bout of inclement weather. How do insects balance their need to enter diapause before winter with their need to consume and store nutrients before diapause? When insects are nutrient limited, do they prioritize allocating nutrients to growth or storing nutrients for diapause? In mammals, dietary fatty acid limitation, particularly unsaturated fatty acids, can decrease the likelihood of entering hibernation and surviving hibernation (e.g., Falkenstein et al., 2001; Frank, 1992). However, the relationships between an insect's diet and diapause phenotypes are much less clear (Short & Hahn, 2023). Understanding the relationship between feeding, growth, and diapause phenotypes is critical to understanding winter survival and thus predicting winter mortality of pests or beneficial insects, especially in the context of climate change (Denlinger, 1991, 2022; Lehmann et al., 2020; Renault et al., 2003; Roberts et al., 2023; Roberts & Williams, 2022; Williams et al., 2015).

A number of previous studies have found that insects at the onset of diapause are heavier than their non‐diapausing counterparts (reviewed by Denlinger, 2022). For example, diapausing adults of the house mosquito, *Culex pipiens*, have a 45% greater wet weight than non‐diapausing adults at the same age (Zhang & Denlinger, 2011). Possibly underlying their elevated weight, diapause‐destined fourth instar mosquito larvae also have an additional day of larval development as compared to their non‐diapause‐destined counterparts (an additional example of extended pre‐diapause development is the green lacewing, *Chrysopa formosa*; Li et al., 2018). Another mechanism that a diapause‐destined insect could use to gain more weight is to increase its digestive efficiency. Diapause‐destined Colorado potato beetles, *Leptinotarsa decemlineata*, have a greater food conversion efficiency than their non‐diapause‐programmed counterparts (Doležal et al., 2007; see also the Fall Webworm, *Hyphantria cunea*, Zhao et al., 2021). However, the relative importance of each mechanism of body mass accumulation is rarely investigated in the context of diapause.

While diapausing insects are often heavier than their non‐diapausing counterparts, there are numerous examples of diapausing insects that are the same weight as, or even lighter than, non‐diapausing insects. For example, in lab‐reared butterflies *Choristoneura fumiferana*, *Papilio polyxenes*, and *Iphiclides podalirius*, diapausing individuals are lighter than non‐diapausing individuals, even though the non‐diapausing larval stage is no longer than the diapausing larval stage (Blau, 1981; Esperk et al., 2013; Harvey, 1961). In multiple populations of the fruit fly *Drosophila montana*, reproductively diapausing adults are also lighter than non‐diapausing individuals, potentially due to accelerated larval development (Salminen et al., 2012). The relationship between diapause and body weight can also be population‐specific. In some populations of the striped ground cricket *Allonemobious fasciatus*, individuals grown from diapausing eggs are heavier than individuals grown from non‐diapausing eggs, while other populations lack this correlation (Mousseau & Roff, 1989). Outside of a few examples, the extent to which feeding rate, feeding duration, and digestive efficiency regulate the body weight of diapausing insects compared to their non‐diapausing counterparts remains under‐investigated.

The effect of feeding duration on the relationship between diapause programming and body weight can be directly tested by removing an insect's food before it completes feeding. Prematurely halting feeding in the lab by removing an insect's diet could simulate the ecologically relevant scenario of an insect depleting its final food source before overwintering. Because diapause‐destined insects must allocate nutrients to diapause preparation in addition to growth and development, abbreviating feeding may more drastically reduce the weight of diapause‐destined insects than non‐diapause‐destined insects. For example, in *Calliphora vicina* blow flies, when larvae are removed from their food before they have finished feeding, diapausing larvae are lighter than their non‐diapause‐programmed counterparts (Saunders, 1997). However, *ad libitum*‐fed larvae that diapause are the same weight as larvae that do not diapause, confirming that abbreviated feeding can reveal the effect of diapause programming on body weight (Saunders, 1997).

In addition to diapause affecting body weight, body weight could also feedback to affect diapause programming. For example, if light or poorly fed insects (i.e., insects with small nutrient stores) are unlikely to survive their winter diapause, will they avert their diapause and instead attempt to produce another generation before the winter? In the longicorn beetle *Psacothea hilaris*, larvae that do not reach a weight that is 330% greater than the minimum viable weight required for metamorphosis will avert their larval diapause (Munyiri et al., 2004). Similarly, in *Calliphora vicina* blow flies, light larvae will also avert diapause (Saunders, 1997). In both cases, experimenters manipulated body weight by abbreviating feeding. Body weight variation is usually induced by manipulating feeding (Munyiri et al., 2004), crowding (Saunders, 1997), or temperature (Nijhout, 2003). All three of these environmental factors could act as cues that the growing season is coming to an end and so may affect diapause likelihood independent of their role in setting body weight. Does body weight affect diapause likelihood, or are body weight and diapause likelihood both downstream of an environmental cue such as food depletion? Experiments that explicitly analyze whether body weight or feeding duration better explain diapause likelihood are needed.

In addition to the frequently observed positive relationship between body weight and diapause likelihood, a longstanding hypothesis in diapause research is that heavier individuals remain in diapause longer (Hahn & Denlinger, 2007, 2011; Saunders, 1997; Wei et al., 2010). The unknown internal mechanism that terminates diapause in many insects remains elusive (Denlinger, 2022; Koštál, 2006), but Hahn and Denlinger (2011) hypothesize that diapause termination becomes more likely as an insect's nutrient stores deplete (see also Zhou et al., 2020). Thus, heavy individuals with large nutrient stores should be able to remain in diapause longer than light individuals with small nutrient stores. In support of this hypothesis, lighter lab‐cultured *C. vicina* larvae spend less time in diapause than heavy larvae (Saunders, 1997). In the field, the first *Bombus huntii* bee queens that emerge in the Wyoming spring are about 15% lighter than queens that emerge 20 days later (Keaveny & Dillon, 2022). The correlation between body weight and diapause duration is consistent with the hypothesis that nutrient stores affect diapause duration. However, another hypothesis that could explain the correlation between body weight and diapause duration is that both heavy and light individuals attempt longer periods of diapause, but only heavy individuals have the nutrient stores needed to survive the longer diapause periods (i.e., survivor bias explains the correlation, Short & Hahn, 2023). However, studies that investigate the association between body weight and diapause duration often cannot measure mortality by body weight interactions because body weight is not estimated prior to overwintering.

**FIGURE 1:**
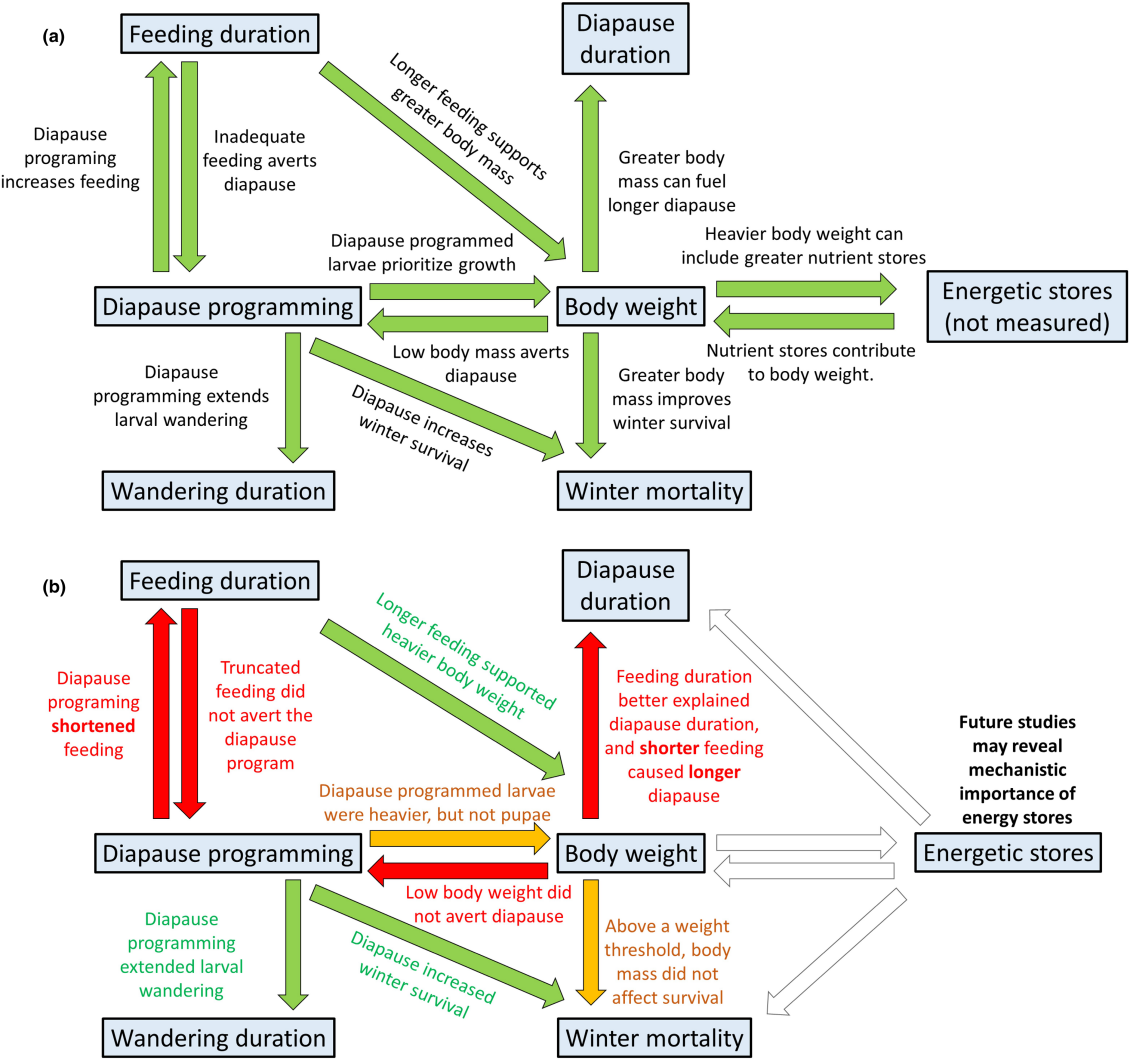
A conceptual map of the relationships between diapause programming, body weight, and diapause‐associated phenotypes depicted based on (a) our hypotheses rooted in the dogma of diapause literature and (b) our results that largely ran contrary to common precepts about the relationship between body mass and diapause. Studies that measure the relationship between body weight and nutrient store accumulation (not measured in this study) under both feeding restriction and diapause‐inducing conditions could test the extent to which diapause programming may affect the relationship between body weight and nutrient storage.

In the present study, we used the relationships between feeding, body weight accumulation, and diapause programming in the flesh fly, *Sarcophaga crassipalpis*, to test a series of long‐standing hypotheses about how diapause affects body weight and vice versa. *Sarcophaga crassipalpis* is a multivoltine fly that can enter a facultative diapause in response to short‐day photoperiods perceived in utero and during larval feeding in the orifices of carrion. First, based on the assumption that diapausing pupae must rely on their nutrient stores to survive the winter, in addition to metamorphic development, we hypothesized that short‐day‐reared larvae would feed and grow more quickly and for a longer duration, therefore achieving a heavier body weight than their non‐diapause‐programmed counterparts (Figure 1a). To test this first hypothesis, we measured the time between larviposition and the onset of wandering behavior, the duration of wandering behavior, larval weight at the onset of wandering behavior, and pupal weight. Second, we hypothesized that individuals with a lighter body weight would more readily abort their diapause program than heavier individuals (Figure 1a). To test the second hypotheses, we prematurely removed larvae from their food to stunt their growth, generating a wide range of larval and pupal body weights in both diapause‐programmed and non‐diapause‐programmed flies. Third, we hypothesized that heavy diapausing pupae are better able to survive winter than both light diapausing pupae and non‐diapausing pupae. Finally, because we expected that heavy diapausing pupae could subsist on nutrient stores longer than light diapausing pupae, we predicted that heavy pupae would remain in diapause longer than light pupae (Figure 1a). To test these final two hypotheses, in a separate experiment, we once again prematurely removed larvae from their food to generate a range of pupal body weights, then kept pupae in simulated winter conditions and measured their survival and the time between larvae exiting winter conditions and completing their development. Surprisingly, our results refute many of our predictions about the relationship between diapause programming, feeding, and body weight (Figure 1b).

## 2 MATERIALS AND METHODS

### 2.1 Colony maintenance

*Sarcophaga crassipalpis* Macquart for our experiments has been cultured continuously for over 15 years, fed a larval diet of *ad libitum* beef liver and an adult diet of *ad libitum* cubed sucrose and dried skim milk powder under a photoperiod of 15 L: 9 D (light: dark) at 25°C. Liver was also included in the diet for the first 7 days after adult eclosion. Adults eclosed and reproduced in metal screen cages of approximately 0.125 m^3^, then larviposited onto raw liver on the 10th day after adult eclosion. Larvae were divided into aluminum foil packets containing ~50 g of beef liver placed in a larger vented container with 1.5 cm of sawdust covering the bottom of the container for pupariation substrate.

For experiments, larvae of the parental generation were collected under the culture conditions above, and then pharate adults were transferred to short‐ (9 L: 15D, 25°C) or long‐day photoperiods (15 L: 9D, 25°C) at the black bristle stage so that adults would emerge into the diapause‐inducing or diapause‐averting photoperiod. The induction phase of flesh fly diapause begins while larvae are in the maternal uterus, a structure through which larvae can sense external photoperiodic cues (Rockey, Miller, & Denlinger, 1989). We subjected mothers to either short‐day or long‐day photoperiods to induce or avert diapause (respectively) in the offspring generation that would be used for experiments. Larvae were reared in aluminum foil packets with *ad libitum* beef liver (50 + 0.9 g of beef liver and 50 larvae per foil packet) within a larger container with sawdust at 20°C under the same photoperiod as their mother. The self‐selected feeding duration experiment was conducted in 2023, but all other experiments were conducted in 2009.

### 2.2 Do diapause‐destined larvae feed longer than non‐diapause‐destined larvae?

To test the extent to which diapause programming affects the duration of feeding, we collected larvae under diapause‐inducing and diapause‐averting photoperiodic conditions and monitored for wandering behavior (a behavioral marker for the cessation of feeding) every 6 h. For this experiment, larviposition was restricted to a 6 h window to improve the accuracy of our age measurements. Aluminum foil packets containing liver were standardized to a base of 3.75 cm × 8.50 cm and a height of 5.00 cm. The onset of wandering behavior, the self‐selected end of a larva's feeding phase, was scored every 6 h by checking for individuals that had exited from the foil packets. Wandering larvae were removed from the sawdust, rinsed with isotonic phosphate buffered saline, and then briefly dried with Kim Wipe® tissue. We then weighed the larvae to the nearest mg. We refer to this weight as “wandering larval weight.” Larvae were then placed into a 5 × 1 cm Petri dish with an approximately 0.5 cm layer of sawdust and returned to their diapause‐inducing or diapause‐averting photoperiods. Wandering larvae were monitored for pupariation every 6 h. Five days after pupariation, once larval‐pupal metamorphosis was complete (Denlinger & Zdárek, 1994), pupae were weighed to the nearest mg. We refer to this weight as “pupal weight.” Observations and weights taken during subjective night were conducted using a red‐light headlamp because red light does not affect the photoperiodic response of the congeneric flesh fly species *Sarcophaga argyrostoma* (Saunders, 1973). Pupae were monitored daily for eclosion. Diapause status was diagnosed based on the duration of the developmental period from pupariation to adult eclosion. Short‐day individuals that extended pupariation‐adult eclosion development beyond the distribution of long‐day‐reared individuals were scored as diapausing.

### 2.3 Do diapause‐destined larvae grow more quickly than non‐diapause‐destined larvae?

To test the extent to which feeding duration affects body weight and diapause likelihood and to test whether feeding duration or body weight better explained variation in diapause likelihood, we removed short‐ and long‐day‐reared larvae from their diet at a range of timepoints prior to their self‐selected termination of feeding. To simulate depleting a food source, forty short‐day reared larvae were removed from their diet at 48, 57, 72, 81, 96, 105, 120, and 129 h after larviposition, and 40 from each long‐day foil packet at the same time points except no individuals were removed at 129 h in the long‐day treatment because most individuals had entered the wandering phase. Larvae were reared at a density of 100 larvae per foil packet with 50 g ± 0.9 g of beef liver that was homogenized in a blender. To estimate weight at removal from diet we briefly washed each larva in Ringer's physiological saline, dried it gently using a Kim Wipe® tissue, and weighed it to the nearest 0.1 mg. We refer to this weight as “removal weight.” We then returned the larvae to their respective photoperiod and temperature in a 150 × 15 mm Petri dish lined with filter paper to be monitored for pupariation daily to determine the duration of the wandering phase. Though we diagnosed this phenotype as wandering duration, we were unable to eliminate the possibility that part of the time spent in locomotion was in search of additional food. Five days after pupariation, all individuals were weighed again. We observed pupae daily for adult eclosion. Individuals that failed to pupariate or complete metamorphosis were recorded so that we could calculate the weight that induced 50% of the population to pupariate or complete metamorphosis (minimal viable weight; Nijhout, 1975). Diapause status was diagnosed as described above.

### 2.4 Do heavy diapausing pupae survive simulated winter better than light diapausing pupae? Do heavy diapausing pupae diapause longer than light diapausing pupae?

To test the extent to which feeding duration affects body weight and survival of our simulated overwintering regime, we removed short‐ and long‐day‐reared larvae from their diet prior to their self‐selected termination of feeding. We also used this dataset to test whether feeding duration or body weight better explained variation in simulated overwintering survival by competing statistical models with each factor against each other. Five hundred short‐day reared larvae were removed from their diet after 72, 81, or 120 h, and 160 or 150 long‐day reared individuals were removed from their diet after 75 or 120 h, respectively. Larvae were monitored daily for pupariation, and 5 days after pupariation, pupae were weighed as described above. After being weighed, 50 short‐day‐reared pupae from each feeding duration were stored at 9°C in constant darkness for a treatment period of 0, 20, 50, 80, 105, 140, 170, 215, 255, or 305 days. Fifty long‐day‐reared pupae from each feeding duration were stored at 9°C in constant darkness for 0, 20, or 50 days. Ten long‐day‐reared pupae that were fed for 75 h were stored at 9°C in constant darkness for 80 days. We stored pupae at 9°C because this temperature is just below their minimum developmental temperature of ~10.7°C (thermal constant threshold; Chen et al., 1987b). However, this temperature is higher than the 6°C temperature that induces rapid cold hardening (Chen et al., 1987a). We refer to this cold storage as “simulated winter.” After simulated winter, flies were placed in diapause‐terminating conditions (15L: 9D at 25°C) and monitored daily for adult eclosion to determine mortality and diapause duration. After 111 days in diapause‐terminating conditions, pupae that had still not eclosed had the pupal cap removed to determine if pupae were dead or appeared to be healthy and in diapause. All pupae that had not eclosed were dead. Diapause was scored as above, with the caveat that simulated winter duration was subtracted from the pupariation‐adult eclosion to calculate developmental duration.

### 2.5 Statistical analysis

To analyze data from all three experiments, we competed hypothesis driven generalized linear models to explain categorical responses like diapause/non‐diapause status and survival, or linear models to explain numeric variables like body weight and feeding duration. We used mixed linear models to analyze the self‐selected feeding duration experiment, including cohort as a random factor. The model that best explained the variance in each response variable was chosen using the Akaike Information Criteria (AIC). If two terms correlated within a model, the variance inflation factor (VIF; summarized in Craney & Surles, 2002) was used to confirm terms could coexist without drastically biasing effect size estimates (models with a VIF > 2.5 were disqualified). Lower AIC values indicated a model that better explained the data, and if the lowest AIC models were within 2 units of each other, the more parsimonious model (i.e., using fewer terms or terms more logically connected) was chosen. Downstream variables were disqualified from explaining upstream variables (i.e., pupal weight could not be used to explain larval weight). Feeding duration was treated as a numeric variable to account for different time points sampled between short‐day and long‐day photoperiods. For winning models, the estimated effect sizes were scaled to the explanatory and the response variables. If an explanatory variable was significant (*p* < .05), we made post‐hoc comparisons between groups using Student's t‐tests, Fisher's exact tests, or Pearson's correlations. Minimal viable weights for pupariation and metamorphosis (the weight that can support 50% pupariation or metamorphosis, respectively) were estimated using the probability distribution generated by a generalized linear model and statistically compared using confidence intervals. In cases where pupal weight was a significant factor, we also compared feeding duration groups post‐hoc because feeding duration underlay the variation we observed in weight. To correct for multiple comparisons, we adjusted *p*‐values using false discovery rate corrections. For our post‐hoc analysis of the effect of photoperiod on larval weight, we adjusted p‐values from all feeding durations together. We also adjusted *p*‐values from all feeding durations together for our post‐hoc analysis of the effect of photoperiod on wandering time and pupal weight. For our post‐hoc analysis of the effect of photoperiod and feeding duration on simulated overwintering survival and diapause duration, we made pairwise comparisons of each feeding duration and photoperiod combination. We adjusted *p*‐values for each simulated winter duration separately. In post‐hoc analysis, we noted a potential threshold of body weight for simulated winter survival. To statistically test for a threshold, we binned weights in 8 mg increments and competed models with binned weights against those with weight as a continuous variable, then used Fisher's test with a false discovery rate correction for post‐hoc comparisons between arbitrary 8 mg weight intervals. We also compared our estimated weight threshold with the *drc* package estimate of weight that causes 50% mortality in simulated winter (Ritz et al., 2015). All statistical analyses were performed using R version 4.2.2 and the code for statistical analysis, including the datasets and all models competed, can be found at https://doi.org/10.6084/m9.figshare.24986235.

## 3 RESULTS

### 3.1 Diapause programming shortens feeding duration and lengthens wandering

To test the extent to which diapause programming affects the duration of larval feeding and wandering behavior, we reared *Sarcophaga crassipalpis* under diapause‐inducing, short‐day photoperiod (9L: 15D) or diapause‐averting, long‐day photoperiod (15L: 9D). We then measured the time between larviposition and the onset of wandering behavior that accompanies the cessation of feeding. We found that diapause programming slightly but significantly accelerated the onset of wandering behavior (Table 1; lmer, *t* = 6.18, *p* < .001, df = 456). However, diapause‐programmed larvae only accelerated their wandering behavior by 1.5 h, ~1% of the time from laviposition to wandering. Age at onset of wandering explained significant variance in wandering larval weight (Table 1; lmer, *t* = −9.99, *p* < .001, df = 457). Larve that wandered at a younger age were significantly heavier than their counterparts that wandered at an older age (Pearson's correlation, *r* = −.336, *t* = −7.62, *p* < .001, df = 457). In other words, delaying wandering behavior was not associated with heavier wandering larval weight. Photoperiod was absent from the model that best explained wandering larval weight. However, because diapause‐programmed larvae accelerated their wandering behavior and younger wandering age was associated with heavier body weight, diapause‐programmed wandering larvae were slightly but significantly heavier than their non‐diapause‐programmed counterparts (an increase of 6 mg or 3%, Student's t‐test, *t* = −2.73, *p* < .01, df = 283).

**TABLE 1:** The models that best explain the phenotypes associated with diapause and body mass.

Model	Term	Estimated effect size	SE	*t* or *z* value	*p*‐value	Sig.
Self‐selected feeding duration ~ Photoperiod (df = 457)	Photoperiod	3.89	0.629	6.18	.0173	*
	Intercept	103	3.14	32.8	1E−09	***
Wandering larval weight ~ Self‐selected feeding duration (df = 457)	Self‐selected feeding duration (h)	−1.46	0.146	−9.99	2E−16	***
	Intercept	348	16.2	21.5	2E−16	***
Removal weight ~ Photoperiod * Feeding duration (df = 413)	Photoperiod	36.9	12.8	2.89	.0041	**
	Feeding duration	1.18	0.0822	21.7	2E−16	***
	Interaction	0.251	0.123	2.04	.0417	*
	Intercept	26.3	8.40	3.14	.0018	**
Wandering duration ~ Feeding duration * Photoperiod (df = 413)	Feeding duration	0.700	0.306	2.29	.0228	*
	Photoperiod	0.0125	0.00197	6.32	6E−10	***
	Interaction	0.0063	0.00294	2.13	.0339	*
	Intercept	6.35	0.202	31.5	2E−16	***
Pupal weight ~ Removal weight * Wandering duration (df = 413)	Removal weight	0.710	0.0413	17.2	2E−16	***
	Wandering duration	0.0626	1.13	1.33	.184	
	Interaction	−0.0011	0.00030	−3.62	.0003	***
	Intercept	−9.91	6.64	−1.49	.136	
Diapause (N/Y) ~ Photoperiod (df = 414)	Intercept	No flies in the long day photoperiod diapaused so effect size, SE, *z*, and *p* are unreliable				
	Photoperiod					
Survival (N/Y) ~ Photoperiod * Sim. winter duration + Pupal weight (df = 1579)	Photoperiod (P)	2.13	0.965	2.20	.0275	*
	Sim. winter duration (W)	−0.253	0.0471	−5.36	8E−08	***
	Interaction (P*W)	0.246	0.0471	5.23	2E−07	***
	Pupal weight	0.0389	0.00348	11.2	2E−16	***
	Intercept	2.81	0.958	2.93	.0034	**
Diapause duration ~ Feeding duration * Storage duration (df = 1093)	Feeding duration	−2.02	0.287	−7.03	6E−12	***
	Sim. winter duration	−0.849	0.188	−4.51	7E−06	***
	Interaction	0.0107	0.00191	5.62	2E−08	***
	Intercept	855	27.5	31.1	2E−16	***

*Note*: All weights are in mg, and all durations are in hours. Estimated effect sizes are scaled to the explanatory and response variables. The explanatory variables are listed in order of their explanatory power. Darker lines are used to distinguish models using the data generated by the three experiments. All competing models can be found in the code at https://doi.org/10.6084/m9.figshare.24986235. * indicates *p* < .05, ** indicates *p* < .01, *** indicates *p* < .001. “df” indicates the degrees of freedom of the residuals.

**FIGURE 2:**
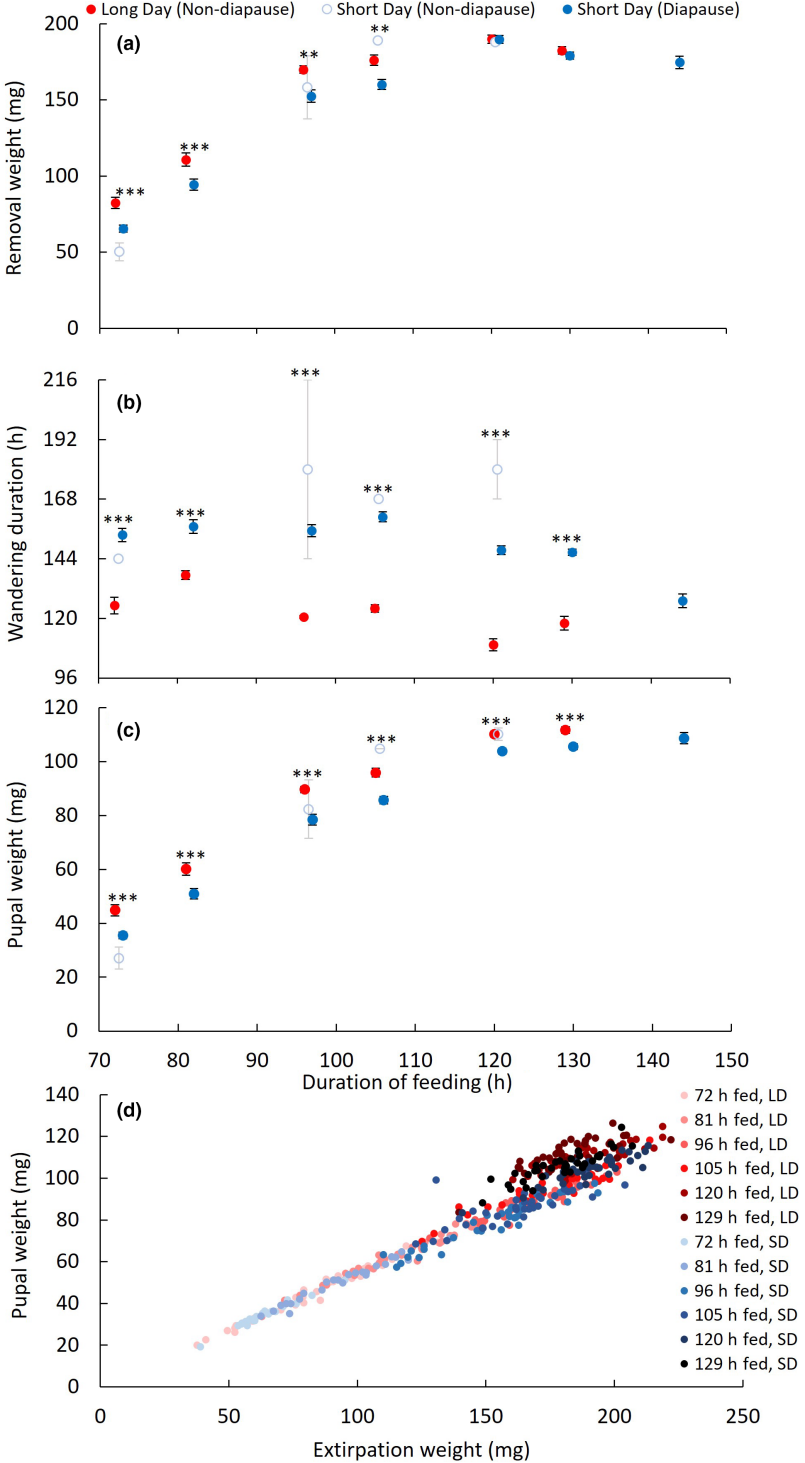
The effects of long‐day photoperiod and extended feeding duration increase pupal weight. Red corresponds to long‐day‐reared flies, empty circles correspond to short‐day‐reared individuals that did not diapause, and blue corresponds to short‐day individuals that entered diapause. For panels a, b, and c, the shared x‐axis displays feeding duration. Bars indicate the standard error. Note that after 81, 129, and 144 h of feeding, all short‐day individuals entered diapause, and in individuals fed 105 h, only one individual reared under short‐day photoperiods did not diapause, so error bars are absent from this data point. Because so few short‐day individuals averted diapause, all short‐day‐reared individuals were grouped together for post‐hoc comparison with long‐day‐reared individuals. Also note that only short‐day‐reared individuals could be removed at 144 h, as long‐day individuals were not tested at this timepoint. Significant post‐hoc differences between photoperiods within feeding durations are indicated with ** or *** (*t*‐test, ** indicates *p* < .01, *** indicates *p* < .001, for all comparisons, df > 35, for all non‐significant comparisons, *p* > .38). (a) Photoperiod, feeding duration, and their interaction explained significant variance in removal weight (Table 1; lm, all |*t*| > 2.04, *p* < .05, df = 413). Removal weight plateaus after 120 h of feeding. (b) Both photoperiod, feeding duration, and their interaction explained significant variance in wandering duration (Table 1; lm, all |*t*| > 2.12, *p* < .04, df = 413). (c) Pupal weight plateaus after 120 h of larval feeding, but at shorter feeding durations, long‐day individuals are heavier as pupae. (d) Removal weight, wandering time, and their interaction explained significant variance in pupal weight (Table 1; lm, all |*t*| > 3.62, *p* < .001, df = 413). Removal weight was positively correlated with pupal weight, regardless of photoperiod or feeding duration (Pearson's correlation, *r* = .975, *t* = 88.7, *p* < .0001, df = 415). Short‐day individuals are blue, and long‐day individuals are red, with deeper color in both photoperiods indicating longer feeding duration.

Diapause‐programmed larvae wandered substantially longer than their non‐diapause‐programmed counterparts. The model that best explained the duration of wandering behavior included photoperiod (lmer, estimated effect size = 3.30 ± 9.67, *t* = 0.341, *p* = .733, df = 454), wandering larval weight (lmer, estimated effect size = 126 ± 36.6, *t* = 3.46, *p* < .001, df = 454), and their interaction (lmer, estimated effect size = 150 ± 48.5, *t* = 3.10, *p* < .01, df = 454). In post‐hoc analysis, diapause‐programmed larvae wandered 72% longer than their non‐diapause‐programmed counterparts, increasing their wandering time by 42 h (see also Figure 2b for extended wandering even when feeding is abbreviated). Heavier larvae wandered for a longer duration than their lighter counterparts (Pearson's correlation, *r* = .328, *t* = 7.42, *p* < .001, df = 457). We tested the correlation between wandering larval weight and wandering duration separately in diapause‐programmed and non‐diapause‐programmed larvae for post‐hoc analysis of the interaction between photoperiod and wandering larval weight. The positive correlation between wandering larval weight and wandering duration was significantly stronger in diapause‐programmed larvae than non‐diapause‐programmed larvae (Pearson's correlation, in diapause‐programmed larvae, *r* = .453, *t* = 8.77, *p* < .001, df = 298, in non‐diapause‐programmed larvae, *r* = .207, *t* = 2.65, *p* < .01, df = 157, 0.358–0.538 95% CI in diapause‐programmed larvae and 0.053–0.351 95% CI in non‐diapause‐programmed larvae). Due to their longer wandering duration, diapause‐programmed larvae pupariated at a significantly older age than their non‐diapause‐programmed counterparts (40 h or 25% older, Student's *t* test, *t* = 25.7, *p* < .001, df = 392).

Wandering larvae lose weight as they clear their gut, transpire water, and use energetic fuel for locomotion. The model that best explained weight lost during wandering included larval wandering weight (lmer, estimated effect size = 0.522 ± 0.017, *t* = 31.5, *p* < .001, df = 454), age at wandering (lmer, estimated effect size = −0.523 ± 0.056, *t* = −8.93, *p* < .001, df = 450), and wandering duration (lmer, estimated effect size = 0.117 ± 0.018, *t* = 6.49, *p* < .001, df = 433). Unsurprisingly, heavier larvae lost significantly more weight during wandering than lighter larvae (Pearson's correlation, *r* = .844, *t* = 33.7, *p* < .001, df = 457). Larvae that wandered at an older age exhibited shorter wandering durations than their counterparts that wandered at a younger age (Pearson's correlation, *r* = −.543, *t* = −13.8, *p* < .001, df = 457). Larvae that wandered for a longer duration lost more weight than larvae that wandered for a shorter duration (Pearson's correlation, *r* = .322, *t* = 7.28, *p* < .001, df = 457). Because diapause programming was associated with both longer wandering duration and wandering at a younger age, diapause‐programmed larvae lost significantly more weight during wandering than their non‐diapause‐programmed counterparts (6 mg more weight lost or 7.5% of larval wandering weight, Student's t test, *t* = 3.65, *p* < .001, df = 240).

The weight of pupae 5 days after pupariation (pupal weight) was unaffected by diapause programming. Though diapause‐programmed larvae had a heavier wandering larval weight, diapause‐programmed larvae lost more weight during their extended wandering behavior, thus reaching the same eventual pupal weight as non‐diapause pupae (average pupal weight was 118 mg, regardless of diapause status, Student's *t* test, *t* = 0.02, *p* = .99, df = 268). The model that best explained pupal weight included wandering larval weight (lmer, estimated effect size = 0.478 ± 0.017, *t* = 28.8, *p* < .001, df = 454), age at wandering (lmer, estimated effect size = 0.523 ± 0.0586, *t* = 8.93, *p* < .001, df = 450), and duration of wandering (lmer, estimated effect size = −0.117 ± 0.0180, *t* = −6.49, *p* < .001, df = 433). Unsurprisingly, wandering larval weight was significantly and positively correlated with pupal weight (Pearson's correlation, *r* = .723, *t* = 22.4, *p* < .001, df = 457). Age at wandering was weakly positively correlated with pupal weight (Pearson's correlation, *r* = .090, *t* = 1.94, *p* = .05, df = 457). Wandering duration had a weak negative effect on pupal weight, but the effect was masked by the strong association between wandering duration and wandering larval weight (Pearson's correlation, *r* = .178, *t* = 3.87, *p* < .001, df = 457). Thus, after *ad lib* *itum* feeding for the duration of larval development, diapausing and non‐diapausing pupae were the same weight.

### 3.2 Diapause programming delays weight gain

To test the extent to which diapause programming affects the course of larval weight accumulation, we reared *S. crassipalpis* under diapause‐inducing, short‐day photoperiods or diapause‐averting, long‐day photoperiods and removed larvae from their diet of raw liver after a series of feeding durations ranging from 48 to 144 h. One hundred and forty‐four hours of feeding surpassed the duration that individuals would eat if they were not removed from their diet. We estimated that larvae had to reach a minimal viable weight of 45.1 mg (95% CI = 38.5 g to 51.7 mg) to successfully pupariate. The minimal viable weight for complete metamorphosis was 74.1 mg (CI spans 66.4 mg to 81.9 mg), significantly higher than the minimal viable weight for pupariation (CIs do not overlap). Neither minimal viable weight for pupariation nor minimal viable weight for complete metamorphosis were detectably affected by photoperiod (our models that best explained pupariation and complete metamorphosis did not include photoperiod). Larvae fed for durations of 57 h or shorter could not reach the critical weight for metamorphosis, regardless of photoperiod, so were excluded from further analysis. At larval feeding durations longer than 71 h (i.e., at feeding durations that could support survival to pupariation), photoperiod, feeding duration, and their interaction explained significant variance in removal weight (Figure 2a; Table 1; lm, all |*t*| > 2.04, *p* < .05, df = 413). In post‐hoc analysis, short‐day‐reared individuals were significantly lighter than long‐day‐reared individuals when fed between 72 h and 105 h, demonstrating that short‐day reared larvae accumulate body mass more slowly than long‐day‐reared larvae (Figure 2a; *t*‐tests, all *t* > 3.26, *p* < .01, df > 55). However, at feeding durations above 105 h, there was no difference in larval weight between the photoperiod treatments, revealing that short‐day larvae grew to the same larval weight as long‐day larvae (Figure 2a; *t*‐tests, all *t* < 0.992, *p* > .32, df > 70).

After larvae voluntarily finish feeding, they become refractory to food cues and begin dispersing and searching for a pupariation site. The duration of the period between larvae being removed from their food and pupariation included this wandering phase. Consistent with our first experiment, photoperiod explained significant variance in wandering duration, but feeding duration and the interaction of photoperiod and feeding duration also explained significant variance in wandering duration (Figure 2b; Table 1; lm, all |*t*| > 2.12, *p* < .04, df = 413). Short‐day‐reared individuals required significantly longer between removal from their diet and pupariation (i.e., wandered longer) than long‐day‐reared individuals at all feeding durations (all |*t*| > 6.53, *p* < .001, df > 35). Well‐fed individuals required significantly less time between removal from their diet and pupariation than poorly‐fed individuals under both short‐day conditions (Pearson's correlation, *r* = −.209, *t* = −2.98, df = 193, *p* < .01) and long‐day conditions (Pearson's correlation, *r* = −.379, *t* = −6.07, df = 220, *p* < .001). Removal weight and the interaction between removal weight and wandering duration significantly explained the variance in pupal weight (Figure 2c,d; Table 1, lm, both |*t*| > 3.62, *p* < .001, df = 413). The explanatory power of wandering duration in our model confirms that short‐day‐reared individuals lost more weight during their extended wandering than long‐day‐reared individuals lost during their brief wandering. In this experiment, even well‐fed (129 ‐h fed) diapausing pupae were slightly but significantly lighter than non‐diapausing pupae (6 mg or 5% lighter, Student's *t* test, *t* = 3.58, *p* < .001, df = 70).

We also tested the extent to which short feeding durations and light body weight could avert the diapause program. Our short‐day photoperiod was so effective in inducing diapause that only 8 out of 195 short‐day‐reared individuals averted diapause, and these non‐diapause individuals were from short and long feeding durations. The model that best explained diapause incidence only included photoperiod (Table 1; glm, absence of diapausing long‐day individuals prevents accurate assessment of effect size, test statistics, or *p*‐values, but post‐hoc Fisher's exact‐test finds diapause is more frequent in short‐day‐reared individuals, χ^2^ = 382.12, *p* < .001, *n* = 417). When feeding duration was forcibly added to a model, feeding duration did not significantly explain the variance in diapause incidence in short‐day‐reared pupae. Short‐day rearing should be considered equivalent to diapause programming throughout our results. However, the few short‐day, non‐diapause individuals we observed are displayed in Figure 2 separately for the reader's convenience. The paucity of non‐diapausing short day individuals clearly shows that diapause‐programmed larvae would not abort the diapause program in response to feeding or body weight cues.

### 3.3 Feeding duration and body weight affect simulated overwinter survival

**FIGURE 3:**
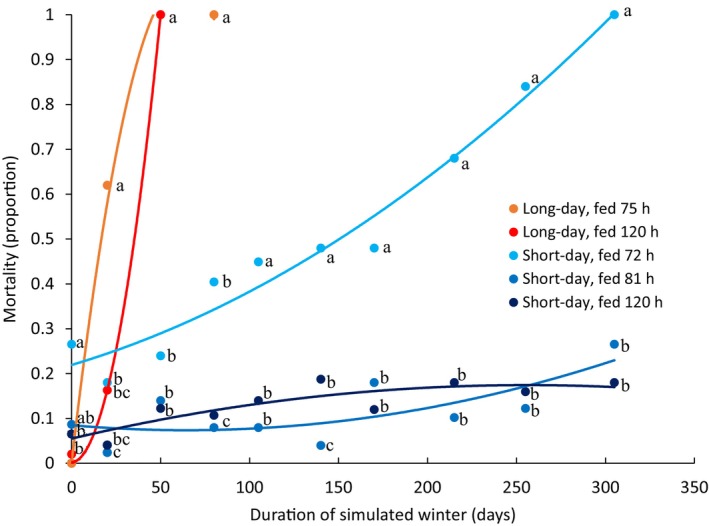
Long‐day‐reared and light individuals died more quickly during the simulated winter. Short‐day‐reared individuals are shown in blue, and long‐day individuals are shown in red, with deeper colors in both photoperiods indicating longer feeding durations. Because each datapoint corresponds to the percentage of mortality across all individuals in each photoperiod‐feeding duration combination, error bars cannot be included in this figure. Storage duration, photoperiod, their interaction, and body weight all explained significant variance in survival at 9°C (Table 1; all |*z*| > 2.20, *p* < .05, df = 937). Significant differences between feeding duration*photoperiod groups within each storage duration are indicated with letters (Fisher's exact test, all *t* > 12.7, *p* < .05, *n* > 94, for nonsignificant comparisons *t* < 12.7, *p* > .06, *n* > 91). Polynomial trend lines are included only to aid in visualizing differences between groups.

**FIGURE 4:**
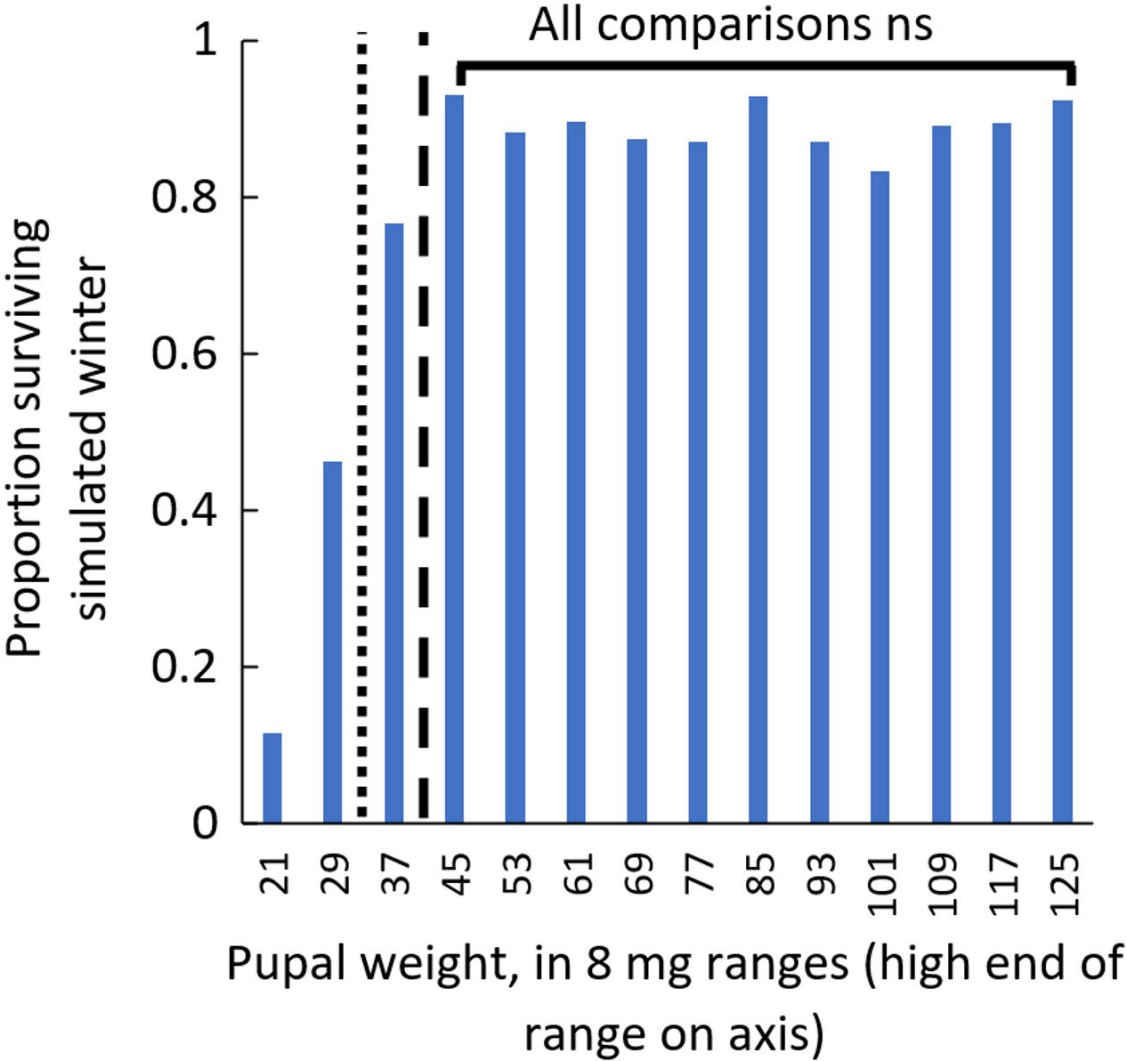
A potential body weight threshold affects the likelihood of simulated winter survival in short‐day‐reared individuals (long‐day‐reared individuals are shown in Figure S2). Proportion surviving is grouped across all storage durations, including 0 days (no simulated winter). Because each bar represents a proportion, no error bars are included, but *n* > 13 for each group. In short‐day‐reared individuals above 0.037 g (dashed line), no body weight groups were significantly different from each other (Fisher's test, all *n* > 66, *t* < 1.29, *p* > .171), suggesting a threshold weight above which body weight does not affect diapause likelihood. Below 0.029 g (dotted line), both body weight groups had significantly less survival than all other body weight groups (Fisher's test, all *n* > 133, *t* > 3.39, *p* < .001).

To test the effects of feeding duration and pupal weight on diapause duration and simulated winter survival, we reared individuals under short‐ or long‐day photoperiods, then stored them at 9°C for up to 305 days. The model that best explained simulated overwinter survival included the duration of storage at 9°C, photoperiod, their interaction, and pupal weight, with pupal weight having the greatest effect size (Figures 3 and 4, Figure S1; Table 1; all |*z*| > 2.20, *p* < .05, df = 1579). Pupal weight outcompeted feeding duration in our model selection (these terms could not coexist within a model because they were colinear). However, feeding duration caused variation in pupal weight, justifying its use in post‐hoc analysis. In post‐hoc analysis, control individuals that were not placed in simulated overwintering had similar mortality regardless of photoperiod or feeding duration, with one exception. Short‐day‐reared individuals that fed for 72 h died more frequently than individuals from either photoperiod that fed for 120 h (Figure 3; Fisher's exact test, both χ^2^ > 7.88, *p* < .01, *n* > 94). In simulated winter, long feeding durations improved survival in both photoperiods. Long‐day‐reared, 120 h‐fed individuals survived longer than long‐day‐reared, 75 h‐fed individuals (Figure 3; Fisher's exact test, χ^2^ > 10.8, *p* < .001, *n* = 98). Similarly, short‐day‐reared, 81 and 120 h‐fed individuals both survived longer than short‐day‐reared, 72 h‐fed individuals (Figure 3; Fisher's exact tests, χ^2^ > 3.84, *p* < .05, *n* > 96). However, short‐day‐reared individuals fed for 81 and 120 h both survived all durations of simulated winter equally well (Figure 3). Short‐day‐reared pupae also survived significantly longer than long‐day‐reared pupae, regardless of photoperiod (Figure 3). Together, our results show that short‐day rearing is more important than feeding duration when explaining survival of all simulated winter lengths, at least within the range of feeding durations used for this experiment. Unsurprisingly, non‐diapause individuals did not survive long periods of simulated overwintering.

Because we found that heavy pupae survived simulated winter better than light pupae, we also tested if heavy pupae better survived simulated winter within each photoperiod and feeding duration. However, feeding duration and pupal weight covary, so they could not coexist within a model. Instead, we tested if surviving pupae had a higher initial pupal weight than dead individuals within each photoperiod and feeding duration combination. In both photoperiods, surviving pupae were heavier than dead pupae only at the shortest feeding duration (Figure S1; *t*‐test, all *t* > 2.36, *p* < .02, df > 45). In both photoperiods, we detected no difference in the pupal weight of surviving and dead pupae at feeding durations longer than 72 h (Figure S1; *t*‐test, all *t* < 1.68, *p* > .11, df > 40). Thus, heavier body weight improved simulated winter survival only in the most poorly fed individuals.

Because we detected that heavier body weight improved simulated winter survival only in the context of very short feeding durations, we hypothesized that above a threshold weight, body weight no longer affects simulated winter survival. To statistically test for a threshold, we competed the model that best explained survival (Table 1; Survival ~ Storage duration * Photoperiod + Pupal weight) with the same model but with pupal weight binned by arbitrary 8 mg increments. Though binned data loses fidelity, it excels at detecting cut‐offs. Treating pupal weight as an ordered categorical variable corresponds to “Is pupal weight above a threshold value?,” while treating pupal weight as a continuous numeric variable corresponds to “Does survival likelihood scale with body weight?” Binned weight outcompeted continuous weight by 94 AIC units, suggesting that survival is much less likely when the weight is below a threshold. In post‐hoc analysis, we examined if a threshold model was competitive within each photoperiod and found that in long‐day individuals, pupal weight better explained survival when it was a continuous variable. However, in short‐day individuals, pupal weight better explained survival when it was binned in arbitrary 8 mg increments. When comparing weights within the short‐day‐reared individuals for post‐hoc analysis, no weights had significantly different survival above 37 mg (Fisher's test, all *n* > 66, *t* < 1.29, *p* > .171). Weights below 29 mg had significantly lower survival than all weights above 29 mg (Fisher's test, all *n* > 133, *t* > 3.39, *p* < .001). Consistent with our finding that survival likelihood changed drastically between 29 mg and 37 mg, we also found that the body weight that causes 50% mortality in short‐day‐reared pupae was 29.4 mg using a logistic regression (CI spans 27.0 mg to 31.8 mg). Above the threshold, survival in diapausing individuals plateaus near 90% regardless of the duration up to 305 days (Figure 4).

**FIGURE 5:**
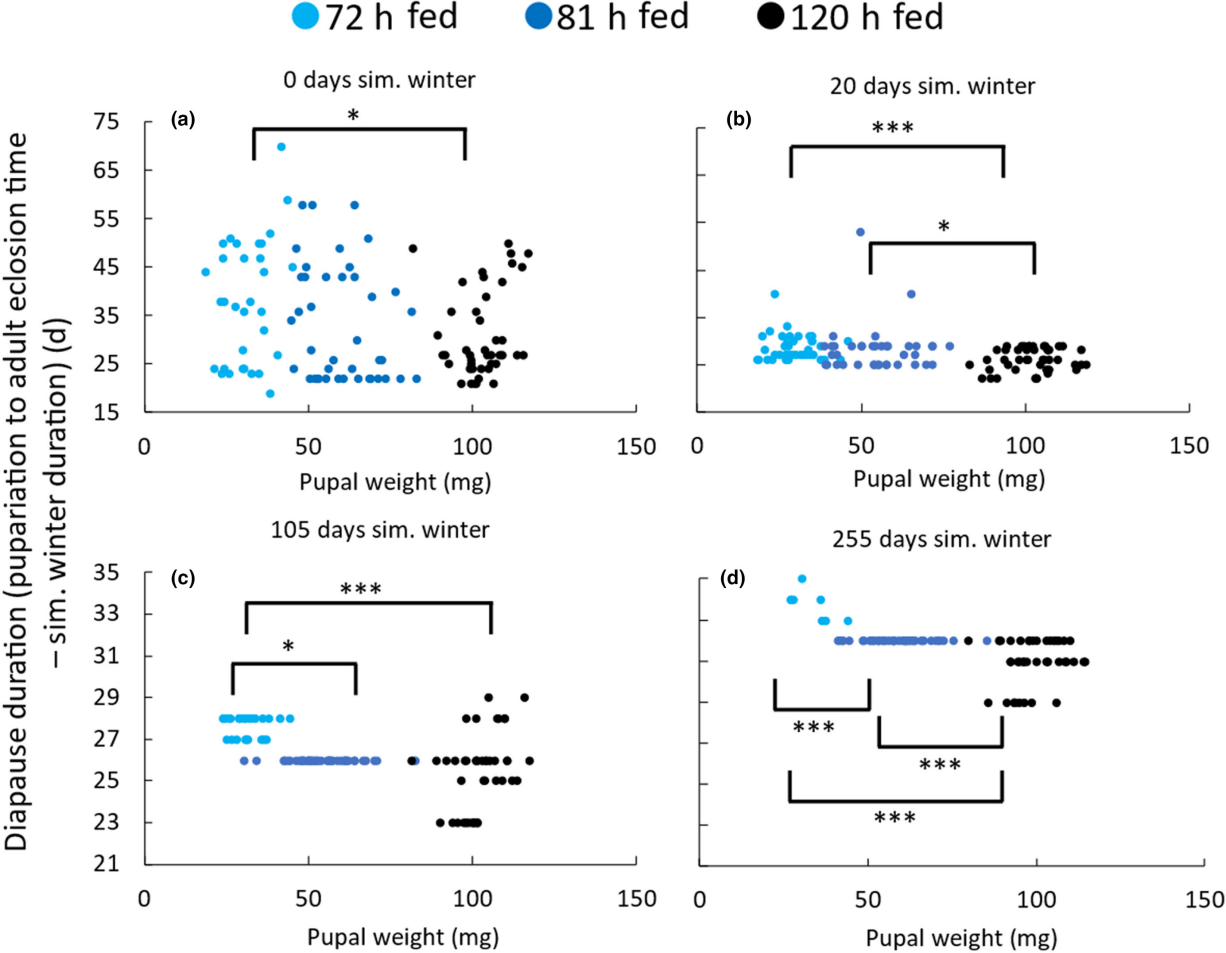
Lighter pupae remain in diapause longer than heavier pupae in all simulated winter durations. The relationships between feeding duration, body weight, and diapause duration are shown in (a) pupae that were not exposed to simulated winter, (b) pupae exposed to simulated winter for the short duration of 20 days, (c) the medium duration of 105 days, and (d) the long duration of 255 days. Significant differences in diapause duration between feeding durations are indicated by * (*t*‐tests, all |*t*| > 2.43, *p* < .03, df > 43) or *** (*t*‐tests, all |*t*| > 4.35, *p* < .001, df > 14). All other comparisons were nonsignificant (*t*‐tests, all *t* < 1.67, *p* > .10, df > 59). Figure S3 shows every simulated winter duration. Diapause duration can only be measured in live individuals, so the number of observations for individuals fed for 72 h dwindles as storage duration increases (see Figure 3).

We hypothesized that heavier pupae would remain in diapause longer than lighter pupae. Feeding duration, storage duration, and their interaction were all present in the model that best explained diapause duration (calculated as time from pupariation to eclosion minus storage duration; Figure 5; Table 1; all |*t|* > 5.62, *p* < .001, df = 1093). Contrary to our expectations, the model including feeding duration outcompeted the model including pupal weight. Also contrary to our predictions, in post‐hoc analysis, short‐day‐reared individuals that fed for 72 h remained in diapause significantly longer after simulated winter than individuals that fed for 120 h across all simulated winter durations (Figure 5; *t*‐tests, all |*t*| > 2.65, *p* < .02, df > 14). Individuals that fed for 72 h remained in diapause significantly longer than individuals that fed for 81 h only when the simulated winter was longer than 20 days (*t*‐tests, all |*t*| > 2.77, *p* < .02, df > 7). Individuals that fed for 81 h remained in diapause significantly longer than individuals that fed for 120 h when simulated winter lasted for 20, 50, and longer than 170 days (*t*‐tests, all |*t*| > 2.43, *p* < .03, df > 39). All other comparisons were nonsignificant (*t*‐tests, all *t* < 1.67, *p* > .05, df > 44). The overall trend was that individuals with longer feeding durations, who were thus heavier, terminated diapause earlier than their briefly fed, lighter, counterparts.

## 4 DISCUSSION

### 4.1 Non‐diapause‐programmed larvae and pupae are heavier than diapause‐programmed larvae and pupae

Insects at the end of their growing season and the onset of winter must make high‐stakes decisions about whether to feed, search for food, or abandon feeding to enter an overwintering state such as diapause. Insects at the end of the season also must decide how to allocate their nutrient resources to cryoprotection, development, and storage. The consequences of this allocation decision are important to an insect's fitness. Insects that feed longer can allocate more nutrients to heavy body weight and large nutrient stores and thus improve their post‐winter fitness (Briegel, 1990; Nielsen et al., 2022). However, insects that remain feeding for too long or allocate inadequate nutrients to cryoprotection may fall to the first bout of inclement weather. Understanding how insects allocate nutrients at the end of the growing season, and the extent to which allocation decisions change if an insect is malnourished may help predict the population sizes of pest insects and beneficial insects as climate change alters the length and severity of winter (Deutsch et al., 2018; Lehmann et al., 2020).

We found that diapausing *S. crassipalpis* pupae were the same weight or lighter than non‐diapausing *S. crassipalpis* pupae, contrary to our expectations and many studies on the effect of diapause on body weight (Figure 1; Denlinger, 2022; Saunders, 1997; Clemmensen & Hahn, 2015). The elevated body weight of long‐day‐reared larvae was first detectable when larvae were removed from their diet prior to wandering and pupariation. At short feeding durations, from 72 to 105 h, long‐day‐reared larvae were significantly heavier at removal than short‐day‐reared larvae. However, by 120 h of feeding, short‐day‐reared larvae had accelerated their weight gain and caught up to their long‐day‐reared counterparts. In our experiment that explicitly measured self‐selected feeding duration, diapause‐destined larvae also accelerated their weight gain near the end of larval feeding, reaching a slightly heavier wandering larval body weight after a shorter feeding phase than their non‐diapause‐destined counterparts. Differences in body weight accumulation between photoperiodic treatments may originate from different feeding rates. Our design did not measure feeding rate because *S. crassipalpis* larvae defecate inside their food, complicating the measurement of uneaten food. In some other insects that have been investigated for the relationship between diapause and feeding rate, feeding rate does not change prior to diapause, only the duration of the feeding period (e.g., in lab‐reared green lacewings, Li et al., 2018). Even if a diapause‐destined larva feeds and ingests the same quantity and quality of nutrients as a non‐diapause‐destined larva, the diapausing larva may need to allocate nutrients to winter hardiness mechanisms. Diapause‐destined larvae may prioritize allocating nutrient resources to surviving winter stresses like cold and desiccation over allocating nutrients to growth or storage. Lee Jr. and Denlinger (1985) found that diapause‐destined *S. crassipalpis* larvae are more likely to survive exposure to −10°C than their non‐diapause‐programmed counterparts, suggesting that diapause‐destined larvae invest in cold hardiness well before pupariation. Taken with our results, Lee Jr. and Denlinger's (1985) findings suggest that larvae may allocate nutrients to cold hardiness early in larval life, at the cost of slowing their growth rate. For example, the cold hardiness molecule glycerol is more abundant in diapausing flesh fly pupae than non‐diapause pupae (Lee Jr. et al., 1987). Thus, though diapause‐destined insects may benefit from being heavier and carrying greater nutrient stores into dormancy, constraints on feeding, nutrient allocation, and developmental duration can make them lighter than their non‐diapause‐destined counterparts in both lab and field settings (Blau, 1981; Esperk et al., 2013; Harvey, 1961; Salminen et al., 2012; Saunders, 1997).

After 105 hours of feeding, diapause‐destined larvae accelerated their growth to reach an equal or slightly heavier weight than non‐diapause‐destined larvae so that after 120 or 129 h of feeding, long‐day‐reared larvae weighed no more at removal from their diet than short‐day‐reared larvae. However, in our first two experiments, short‐day‐reared larvae wandered significantly longer than long‐day‐reared larvae before pupating. Thus, diapause‐destined pupae were always lighter than or of the same weight as non‐diapause‐programmed pupae. We found that short‐day‐reared larvae wandered ~2 days longer than long‐day‐reared larvae at all feeding durations, consistent with findings in *Sarcophaga bullata* (Denlinger, 1972). Though larvae removed from their diet early may have spent time searching for food before wandering, we found that diapause‐inducing photoperiod extended wandering regardless of feeding duration, suggesting hunger was not responsible for extended wandering. We found that diapause‐destined larvae lost more body weight during their extended wandering phase than non‐diapause‐programmed larvae lost during their brief wandering phase. Larvae that wander longer are likely to suffer greater metabolic drain and water loss than briefly wandering larvae. Diapausing pupae may pay this price in body weight because they must remain in their pupariation location for months, in contrast to non‐diapausing pupae that remain in their pupariation location for only a few weeks. Wandering longer may increase a larva's chance of finding a desirable pupariation site that is in a protected microclimate and reduce risk from predators and pathogens (Denlinger, 1972, 2022). We hypothesize that the availability of high‐quality overwintering sites and the cost of overwintering at a low‐quality overwintering site affect the relationship between diapause programming, wandering duration, and body weight (Roberts et al., 2021; Turnbull et al., 2023).

Overall, we found that diapausing pupae were the same weight or lighter than non‐diapausing pupae, contrary to the general trend that diapausing insects are heavier than their non‐diapause‐programmed counterparts (Figure 1; Denlinger, 2022). Combined with the growing faction of studies that have found diapausing insects are lighter than non‐diapausing insects (Blau, 1981; Esperk et al., 2013; Harvey, 1961), our findings emphasize that the relationship between body weight and diapause is complex. We propose that the relationship between body weight and diapause is governed by many ecological factors, such as depleted food sources and how much time remains until inclement weather conditions begin (as estimated by the insect based on photoperiod and temperature). Gotthard et al. (1999) hypothesized that caterpillars use photoperiod to estimate the proximity of harsh winter conditions and accelerate their growth to diapause before seasonally inclement weather begins, albeit at a lighter weight. They tested the relationship between photoperiod, growth rate, development rate, and terminal body weight in a univoltine population of diapausing Nymphalid butterflies. When reared under short photoperiods, caterpillars slightly accelerate their growth rate and dramatically accelerate their developmental rates, leading to a lighter diapausing body weight than the diapausing body weight of caterpillars reared under longer photoperiods. We used a very short day as our diapause‐inducing photoperiod. We hypothesize that in our experiments, extreme short‐day photoperiods may have indicated imminent winter, causing larvae to prioritize early pupariation over body weight. Further work done across a range of diapause‐inducing photoperiods is needed to test this hypothesis in *S. crassipalpis*.

We also tested the long‐standing hypothesis that lighter individuals are more likely to abort their diapause program in favor of non‐diapause development than heavier individuals. We found that short‐day‐reared individuals would not abort their diapause program, regardless of feeding duration or body weight. Our finding that diapause programming is refractory to feeding duration and body weight runs contrary to many studies that have reported that diet composition or malnutrition can affect diapause likelihood (reviewed in Denlinger, 2022; Short & Hahn, 2023). As above, we suggest that the relationship between body weight and diapause must be carefully considered within the natural history of the animal.

### 4.2 Longer feeding durations shorten diapause, despite heavier body weight improving survival of long simulated overwintering

We tested the long‐standing hypothesis that heavy diapausing insects can better survive the winter than light or non‐diapausing insects. Consistent with this hypothesis, we found that short‐day programming and heavy body weight improved survival in our range of simulated winter durations. Our findings are consistent with a wealth of literature showing that photoperiod (Bale & Hayward, 2010; Denlinger, 2022; Teets & Denlinger, 2013) and body weight (Renault et al., 2003; Treanore & Amsalem, 2020) affect overwintering survival. However, our findings are unique in that we detected a body weight threshold, above which body weight was not correlated with simulated winter survival. Feeding and weight thresholds are well characterized for their role in development (Fronstin & Hatle, 2008; Grunert et al., 2015; Mirth & Shingleton, 2012), but are rarely used in the context of overwintering survival. In the context of climate change, a body weight threshold governing winter survival could obscure warning signs that insects are becoming phenologically desynchronized from their food sources. Insect populations that become mildly desynchronized from their food sources may remain above their weight threshold and thus maintain normal population levels. However, once desynchronization becomes extreme, insects could fall below their weight threshold, and populations may rapidly decline.

Do long‐day‐reared or underweight pupae survive simulated winter more poorly than short‐day‐reared or heavier pupae because they deplete their nutrient stores? Though our simulated winter was cold enough to prevent development, it was likely warm enough to generate substantial metabolic demand over time. In the apple maggot fly, *Rhagoletis pomonella*, the metabolism of diapausing pupae is lower than non‐diapausing pupae, even when flies are chilled to 4°C (Toxopeus et al., 2021). We hypothesize that our non‐diapausing *S. crassipalpis* pupae had a higher metabolic rate at 9°C than their diapausing counterparts (as they do at 25°C; Denlinger et al., 1972), and thus depleted their nutrient stores more quickly, causing mortality. Consistent with the hypothesis that nutrient store depletion causes mortality, short‐day‐reared individuals that fed only 72 h suffered higher mortality as simulated winter duration increased (Figure 3). Short‐day‐reared individuals that fed for a longer duration, and presumably had larger nutrient stores survived well even at the longest simulated winter duration that we tested. However, we did not measure nutrient store size in our experimental design, preventing us from attributing weight accumulation to additional nutrient stores. Study designs that measure the accumulation and depletion of nutrient stores like glycogen, triacylglycerol, and storage proteins during the diapause program, especially in the context of food restriction, are needed to explicitly test the extent to which any particular class of nutrient stores affects entry into, exit from, and survival of the diapause program (for review of current evidence, see Roberts et al., 2023).

An alternative hypothesis that could associate body weight, photoperiod, and cold survival is that water loss causes mortality in stored individuals and that long‐day‐reared and underweight individuals lose water more quickly. Diapausing flesh fly pupae lose water more slowly than non‐diapausing pupae (Yoder & Denlinger, 1991), in part because diapause‐destined larvae add an additional layer of cuticular wax to their puparia (Yoder & Moreau, 1994). Also, heavier individuals generally have a lower surface area to volume ratio than lighter individuals, and thus lose water more slowly (Kühsel et al., 2017). Our study design did not include measuring water loss rate, but we hypothesize that water loss could cause mortality during a simulated winter.

We also tested the long‐standing hypothesis that heavier insects remain in diapause longer than lighter insects (Hahn & Denlinger, 2007, 2011; Saunders, 1997; Wei et al., 2010). We predicted that individuals that fed longer would have more resources to survive overwintering, allowing them to remain in diapause longer, prolonging the development between pupariation and adult eclosion compared to individuals with a shorter feeding duration. However, we found that well‐fed individuals needed ~2.3 fewer days between the end of simulated overwintering and adult eclosion than their poorly‐fed counterparts. Why might better‐fed individuals have shorter pupariation to adult eclosion development and presumably shorter diapause duration than poorly‐fed individuals? In the field, shortening the time between the end of winter and adult eclosion would allow a fly to emerge earlier in the spring. One ecological explanation for delayed diapause termination in poorly‐fed individuals is that early emerging, well‐fed individuals can survive off nutrient stores if they emerge before food sources are readily available. Poorly fed individuals lack large nutrient stores, so they cannot risk emerging early and must remain in diapause longer. We hypothesize that the phenology of an insect's food and oviposition sites underlies the relationship between body weight and diapause duration. More specifically, we predict that underweight insects with patchy food availability in the spring will delay their diapause termination, but undersized insects with predictable food availability will accelerate their diapause termination. Most studies have found that body weight positively correlates with diapause duration (Hahn & Denlinger, 2007, 2011), especially in the length of multiyear diapause (i.e., extended diapause to reach future growing seasons, Danforth, 1999; Matsuo, 2006). Several studies have shown that insects emerging after a multiyear diapause are heavier than insects that only diapause for one winter (Danforth, 1999; Matsuo, 2006). We have suggested the correlation between body weight and diapause length may be driven by the death of lighter individuals (i.e., survivor bias; Short & Hahn, 2023). Our finding that lighter individuals indeed die more frequently during simulated winter supports our hypothesis, as do other studies showing underweight individuals survive simulated winter more poorly (e.g., Beekman et al., 1998; Knapp & Řeřicha, 2020). However, our design did not measure nutrient store quantity in relation to body weight. Study designs that measure nutrient stores and body weight are needed to test the extent to which nutrient stores affect diapause induction, diapause initiation, diapause duration, and survival of the overwintering period (for review of current evidence, see Roberts et al., 2023).

Overall, our study produced several findings that ran contrary to our expectations and common conceptions in the literature on diapause and body weight (Figure 1; reviewed by Denlinger, 2022). We predicted that diapausing individuals would be heavier than non‐diapausing individuals, but across all feeding durations, we found that diapausing individuals were lighter than their non‐diapausing counterparts. This suggests that diapause‐programmed larvae may have prioritized nutrient allocation to stress hardiness over allocation to growth. Contrary to our predictions, we found that poorly‐fed individuals either stayed in diapause longer or extended their post‐diapause development time compared to their well‐fed counterparts. Overall, we found that short‐day programming and sufficient feeding support overwintering survival, but the relationship between feeding, body weight, and overwintering ability is more complex. The nuances of our findings suggest that researchers should evaluate an insect's pre‐diapause allocation decisions and the costs of those decisions in a more complete ecological framework that explicitly considers the proximity of winter, the availability of food sources before and after the winter, and the availability of high‐quality overwintering sites. This ecological framework may also prove useful in examining the duration of mammalian hibernation as well as other forms of dormancy.

Our finding that winter survival is no longer affected by weight once an insect passes a weight threshold may help predict how climate change may affect pest and beneficial insect populations. Deutsch et al. (2018) predict that insect pest damage to crops in temperate zones will increase by 10–25% per degree Celsius of warming, largely because overwintering survival will improve. However, climate change may also alter the timing and abundance of insect food sources, causing phenological desynchronization between insects and their hosts that forces insects to prematurely terminate feeding (Lehmann et al., 2020; Williams et al., 2015). Premature termination of feeding could cause a lighter body weight and poor winter survival, thus mitigating the increased pest pressure caused by global warming. However, we found that, above a body weight threshold, winter survival is unaffected by weight. A mild phenological mismatch between insect pests and their hosts is unlikely to push many insect pests below their body weight threshold, and thus we predict such asynchrony will do little to mitigate the elevated pest pressure caused by climate change. On the other hand, overwintering mortality of beneficial insects may only increase once phenological mismatch is so severe that insects fall beneath their body weight threshold, potentially masking early warning signs that beneficial insects are becoming phenologically mismatched from their hosts. Understanding that diapausing insects are not always heavier than non‐diapausing insects and that body weight does not always correlate with winter survival should inform climate change scenarios of increased pest pressure and beneficial insect decline. Testing for body weight thresholds in other dormancy states, like mammalian hibernation or summer aestivation, may be critical to understanding how climate change will alter overwintering mortality and thus species ranges.

## AUTHOR CONTRIBUTIONS

**Clancy A. Short:** Conceptualization (supporting); data curation (equal); formal analysis (equal); visualization (lead); writing – original draft (lead); writing – review and editing (equal). **Daniel A. Hahn:** Conceptualization (lead); funding acquisition (lead); investigation (lead); methodology (lead); project administration (lead); supervision (lead); writing – review and editing (equal). **Jared L. Walters:** Conceptualization (supporting); investigation (equal); methodology (supporting).

## CONFLICT OF INTEREST STATEMENT

The authors have no conflict of interest to declare.

## Supporting information


Figures S1–S3.

## Data Availability

We have made our data and the code used to analyze our data publicly available at https://doi.org/10.6084/m9.figshare.24986235.
