# Supplementary material for: Bigger isn't always better: Challenging assumptions about the associations between diapause, body weight, and overwintering survival
Source: Ecol Evol. 2024 Jun 3;14(6):e11511. doi: 10.1002/ece3.11511 (PMC11148123; doi:10.1002/ece3.11511)
Supplement: Supplementary file 1 — Figures S1–S3. [file ECE3-14-e11511-s001.docx]

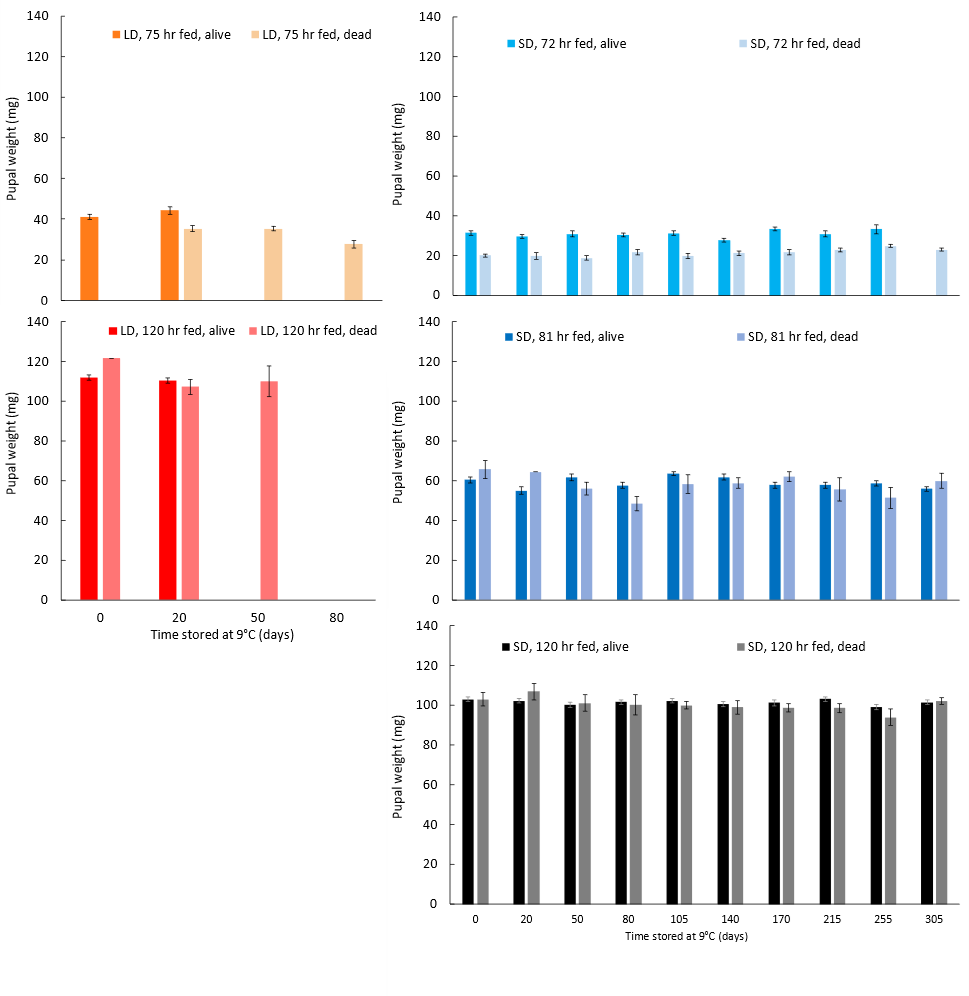


***

*

***

***

***

***

***

***

***

***

na

Figure S1. Long-day photoperiods and the combination of short feeding duration and light body weight decrease survival. The relationship between pupal weight and survival during simulated winter is shown separately for (A) long-day reared individuals fed 75 hours, (B) long-day reared pupae fed 120 hours, (C) short-day reared pupae fed 72 hours, (D) short-day reared pupae fed 81 hours, and (E) short-day reared pupae fed 120 hours. Each bar shows average weight ± standard error. Error bars are absent if only one pupa died. Within each feeding duration, significant differences in pupal weight between individuals that did and did not survive are shown above bars with * indicating p<0.05 (t-test, t>1.39, n=49), *** indicating p<0.001 (t>3.59, n>46), and “na” indicating that a t-test could not be conducted because fewer than 2 individuals died. For all non-significant comparisons p>0.11 (t<2.01, n>49).

34

59

33

13

4

0

2

2

10

22

53

49

24

7

Figure S2. No body weight threshold was detected in simulated-winter survival in long-day reared individuals (short-day reared individuals are shown in Figure 4 of the manuscript). Proportion surviving is grouped across all storage durations, including 0 days (no simulated winter). Because each bar represents a proportion, no error bars are included, but n is included above each group. Because long-day reared individuals were only fed 75 and 120 hours, coverage over all weights is poor, making this dataset ill-suited to detecting a threshold.


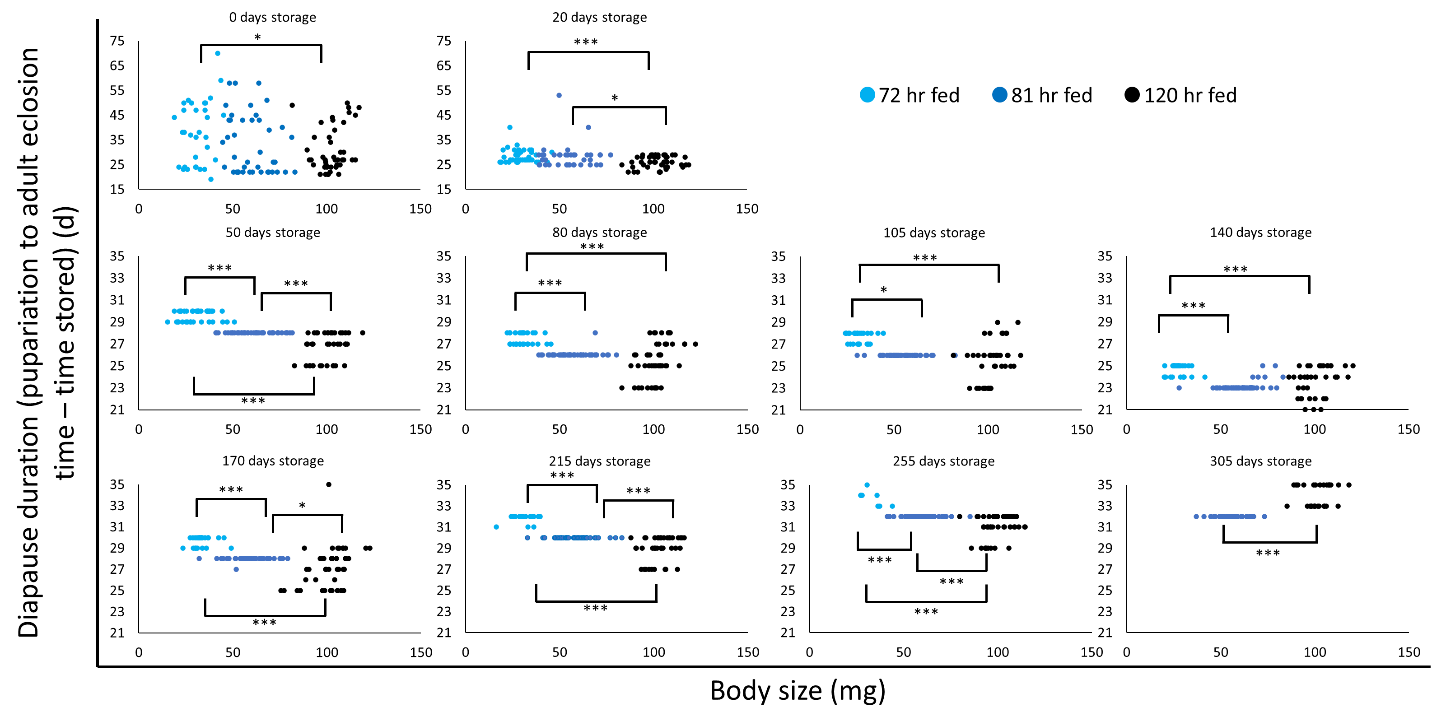


**J**

**I**

**H**

**G**

**F**

**E**

**D**

**C**

**B**

**A**

Figure S3. Smaller pupae remain in diapause longer than larger pupae in all simulated-winter durations (corresponding to Figure 7 in the manuscript). The relationships between feeding duration, body weight, and diapause duration are shown in (A) pupae that were not exposed to simulated winter, (B) pupae exposed to simulated winter for the shortest duration of 20 days, (C) the short duration of 50 days, (D) the medium duration of 80 days, (E) the medium duration of 105 days, (F) the medium duration of 140 days, (G) the medium duration of 170 days, (H) the long duration of 215 days, (I) the long duration of 255 days, and(I) the longest duration of 305 days. Significant differences in diapause duration between feeding durations are indicated by * (t-tests, all |t|>2.43, p<0.03, df>43) or *** (t-tests, all |t|>4.35, p<0.001, df>14). All other comparisons were non-significant (t-tests, all t<2.06, p>0.05, df >44). Diapause duration can only be measured in live individuals, thus the number of observations for individuals fed for 72 hours dwindles as storage duration increases (see Figure 2).
